# Violent victimization and health service utilization in a forensic psychiatric context: a comparison between offenders with mental disorders and matched controls

**DOI:** 10.1186/s12888-017-1251-0

**Published:** 2017-03-11

**Authors:** Mats Persson, Henrik Belfrage, Marianne Kristiansson

**Affiliations:** 1 Department of Clinical Neuroscience, Karolinska Institutet, Stockholm, Sweden; 2 Vadstena Forensic Psychiatric Hospital, Vadstena, Sweden; 3 Department of Medical and Health Sciences, Linköping University, Linköping, Sweden; 4 Department of Forensic Psychiatry, National Board of Forensic Medicine, Stockholm, Sweden

**Keywords:** Victimization, Violence, Forensic psychiatry, Mental disorder

## Abstract

**Background:**

Offenders with mental disorders constitute a particularly exposed group in society, with high rates of morbidity, mortality, and social deprivation. Often thought of primarily as perpetrators, these individuals may also be subjected to violence. Previous research indicates that violent victimization during lifespan is a risk factor for violent perpetration among psychiatric patients, but victimization studies on the group of offenders with mental disorders are scarce. Health services are pivotal to this group, but although most individuals do utilize these services, their vulnerability seems to remain. This study aimed at exploring the rates of victimization and health service utilization, including perceptions of unmet health care needs, among offenders with mental disorders.

**Methods:**

Two hundred detainees undergoing a forensic psychiatric evaluation in Stockholm were asked about violent victimization and health service utilization. Each detainee was compared with three controls from the general population, matched regarding age, sex, and occupation.

**Results:**

Victimization during the past year was reported by 52.3% of the detainees and 11.1% of the controls, with a corresponding risk ratio of 8.2. Health service utilization during the past three months was reported by 47.7 and 23.7%, respectively (risk ratio 2.0); and unmet health care needs by 42.2 and 16.7%, respectively (risk ratio 3.4). There was no distinct association between victimization and health service utilization among detainees.

**Conclusions:**

Offenders with mental disorders are at great risk of being victimized, and they experience impediments to receiving requisite health care. A possible way to reduce victimization and improve health service utilization may be to establish interdisciplinary yet specialized health centers with outreach teams but without complicated referral procedures.

## Background

Offenders with mental disorders constitute a particularly exposed group in society. They often have multiple psychiatric diagnoses [1], somatic comorbidity [2], and addiction [3]; mortality rates are high [4–6] and social deprivation is widespread [7]. Often thought of primarily as perpetrators, these individuals may also be subjected to violence to a large extent, as some of the aforementioned problems also are associated with violent victimization [8]. Furthermore, recent violent victimization may be a risk factor for violent perpetration among persons with severe mental illness comprising schizophrenia, bipolar disorder, and major depression [9]. The health care services are pivotal to this group, but although most individuals utilize both somatic health services [2] and mental health services [10], much of their vulnerability seems to remain. Based on these findings, we wanted to conduct a study on victimization and health service utilization, including perception of unmet health care needs, among offenders with mental disorders.

### Violent victimization

Violent victimization is ubiquitous, and violence has been described as a global health problem by the World Health Organization [11]. According to the 2004–5 International Crime Victims Survey, the annual victimization rate was 0.6% for sexual assault and 3.1% for other assaults and threats, but much of the problem is hidden, as only around a third of the assaults are reported to the police [12]. Consequently, interview surveys demonstrate higher rates; 6.2% of adults in Sweden reported that they were subjected to violence or threats in a twelve-month period [13].

Although universal, violent victimization is not evenly distributed in the population. Well-known demographic risk factors are young age, male sex, unemployment, unmarried state, and poverty [14]; other important risk factors are homelessness [15, 16] and substance abuse [17]. Based on these and other factors, theoretical frameworks have been formed. Three of the most prominent theories are the lifestyle theory, the routine activities theory [18], and the victim precipitation theory. According to the *lifestyle theory*, an individual’s demographics, such as age and sex, interact with role expectations and social constructs, which in turn determines the lifestyle and in that way also the exposure and risk of victimization [19]. The *routine activities theory* suggests that victimization depends on the presence of a potential offender and suitable victim, and on the absence of capable guardians; this is influenced by daily routine activities such as employment, leisure activities, and socializing [20]. Finally, the *victim precipitation theory* posits that victims contribute or sometimes even instigate to the violent acts being committed [21].

These theories may explain why violent victimization is even more widespread in certain groups. One risk group consists of persons with severe mental illness, where a recent review states an annual prevalence of 6.4–56% [8], and a lifetime prevalence of over 90% for traumatic events has been reported [22, 23]; victimization is also associated with greater current symptomatology [24]. Indeed, research indicates that persons with severe mental illness are more likely to be victims than perpetrators of violence [25]. However, offending is a risk factor for victimization [26], which applies also to persons with mental disorders [27], in whom the presence of offending is associated with an 11-fold increase in victimization [28]. Victimization may in turn be a risk factor for offending: a Swedish registry-based study showed that persons diagnosed with schizophrenia spectrum disorders or bipolar disorder were significantly more likely to commit violent crimes if they had been subjected to violence in the previous week compared with earlier periods for the same individuals, with adjusted odds ratios ranging from 7.6 to 12.7 [29]. There appears to be a paucity of studies of violent victimization using study samples consisting of offenders with mental disorders, but higher rates are reported for inmates with dual disorders [30] or any mental disorder [31] than for other inmates during incarceration.

### Health service utilization

In general, the state of public health in the Western world is satisfactory, and to a certain degree, this may be due to the highly accessible health care services in the region. For example, 61.0% of American adults reported excellent to very good health, and 80.3% undertook at least one visit to a health care professional in a twelve-month period [32]; whereas 80.2% of Swedish adults report good health, and 35.3% undertook at least one visit to a doctor in a three-month period [13]. However, in some western countries more than 10.0% of adults report unmet health care needs [33].

Unfortunately, the general state of health among individuals with mental disorders is poorer. For example, when persons with schizophrenia are compared with the general population, diabetes and cardiovascular diseases are at least twice as common [34], the all-cause standardized mortality ratio is 3.6 [35], and the expected lifetime is reduced by 15–20 years [36]. The general state of health is poor also among offenders [37]: the psychosis prevalence is 3.6% and the major depression prevalence is 10.2% among prisoners [38], equivalent to a two-fold to four-fold excess in comparison with the general population [39]; and around 40% of American prisoners suffer from chronic physical medical conditions [40]. The findings from these two populations indicate that the general health state is especially poor among offenders with mental disorders, even though they utilize a considerable amount of health care services [2, 10]. This imbalance suggests the occurrence of unmet health care needs, but studies in the field are scarce. There may be an association between violent victimization and health care utilization, in so far as the former is reduced by the latter to some extent [41].

### Aims and hypotheses

This study aimed at describing the extent of violent victimization and health service utilization in a Swedish forensic psychiatric sample, and at comparing it with controls from the general population. We hypothesized that offenders with mental disorders would have higher rates of (1) violent victimization, (2) health service utilization, and (3) unmet health care needs than the general population. Furthermore, we hypothesized that these three rates would be higher for offenders with severe mental disorders than for offenders with non-severe mental disorders (4, 5, 6). Finally, we wanted to explore the association between violent victimization and health service utilization among offenders with mental disorders.

## Methods

### Study design

In the present study, self-reported rates of violent victimization, health service utilization, and unmet health care needs for offenders with mental disorders were compared with the corresponding rates for controls. Controls were matched for age span, sex, and occupation.

### Study sample

Our study sample consisted of 200 detainees between 18 and 60 years old, undergoing a major forensic psychiatric evaluation at the Department of Forensic Psychiatry in Stockholm. The sample size was determined by a power calculation. Based on a previous study of similar design [42], we wanted to detect a proportion difference of ten percentage points between the study sample (15%) and the control group (5%) regarding exposure to violence that caused a visible injury. With 188 in each group, there was 90% power (α .05, two-sided) to detect this difference, but to allow for attrition, we included 200 detainees. To reach this number, 286 detainees were approached, of whom 65 declined, 15 left the department before the interviews were completed and 6 were too psychotic to be able to give their informed consent.

We divided the study sample into two sub-groups: one consisting of detainees with severe mental disorders according to the forensic psychiatric evaluation, and one of detainees with non-severe mental disorders. In Sweden, severe mental disorder is a legal rather than medicinal construct, which came about when the Swedish penal code was modified in 1992. According to the bill that preceded the legislative change, the construct consists of psychotic conditions with delusions, hallucinations or confusion; depression with suicidal ideation; personality disorders with recurrent psychotic episodes or severe compulsive behavior; and serious impairment of mental functioning due to dementia, brain damage or intellectual disability.

### Control group

The control group was provided by Statistics Sweden, a government agency that produces statistical data. Each year since 1975, the agency has conducted a survey called SILC, the Swedish living conditions survey [43], covering areas such as health, employment, and security. This survey is carried out by means of telephone interviews with a random sample consisting of 12,000-13,000 persons. Respondents have to be over the age of 16 and be included in the Swedish population registry, otherwise there are no exclusion criteria. This means that the respondents can be located anywhere in the community and engage in any activity.

From this SILC sample, we obtained three matched controls for each offender; hence, the control group consisted of 600 individuals. More than three controls per offender would probably not have improved efficiency [44, 45]. To handle the problem of confounding, we chose to carry out a regular matching. This method mimics a blocked randomized experimental design, and as no observations are pruned, it is powerful and efficient. Matching creates a sample of controls that is not entirely representative of the population as a whole [46], so the number of matching variables should be restricted. Since age, gender, and employment are important factors related to victimization [14], the controls were matched by ten year age span (16–25, 26–35, 36–45, 46–55, and 56–65 years), sex, and occupation (employed, unemployed, student). Another possible matching variable, marital status, was excluded as forms of intimate relations are rapidly changing in society.

### Measures

There are no clear-cut definitions of violent victimization and health care utilization. In this study, we based the constructs on six SILC questionnaire questions [47] that both detainees and controls were asked. These constructs are described in Table 1.

**Table 1:** Definitions of indicators in the SILC questionnaire

Victimization	Health service utilization
Since MONTH YEAR (the past twelve months), have you been subjected to…	Have you, during the past three months, because of own illness visited a doctor?
…violence that led to injuries that forced you to seek medical care or dental care?	Have you, during the past twelve months^a^, ever considered yourself in need of medical care but did not seek medical care?
…violence that led to visible marks or injuries that did not require medical care or dental care?	
…violence that did not lead to visible marks or injuries?	
… threats of violence or other threats that were so serious that you were frightened?	

*SILC* Swedish living condition surveys. ^a^Three months in the 2006 and 2007 versions of the SILC questionnaire

For the detainees, the 2006 version of SILC was used for practical reasons; the authors were well versed in its use. For the controls, the 2006–2013 versions were used. The versions differed in one considerable respect: the 2006 and 2007 versions ask for unmet health care needs during the past three months, while the other versions use a twelve-month frame.

### Procedure

While undergoing a major forensic psychiatric evaluation in Stockholm, the detainees were asked the six SILC questions in Table 1. These interviews took place between August 2011 and July 2013 and were conducted by a forensic psychiatrist (the first author) or an experienced research assistant. Demographic, clinical, and forensic data were gathered from the detainees themselves and from their case files. The controls were asked the SILC questions over the telephone by professional interviewers from Statistics Sweden between 2006 and 2013, this time frame was necessary to get a sufficient quantity of controls. These interviews were part of the regular SILC surveys performed by the agency. Additional interviews for inter-rater reliability testing were not considered necessary, as the questions were read verbatim.

During the time at risk, i.e. the three to twelve months prior to the study enrolment, the participants could reside at any location. As the victimization occurred during this time, it could take place anywhere. The health service utilization also occurred during the time at risk. Health services in Sweden are extensive and funded primarily through taxation; only a small and limited fee is charged.

### Statistical analyses

All statistical analyses were conducted using SPSS, version 22. To compare groups, we used *χ* ^2^ tests and Fisher’s exact tests for categorical variables, and the Mann–Whitney test for the age variable, that was not normally distributed. When calculating risk ratios, conditional logistic regressions (in SPSS: Cox regressions with time as an arbitrary constant) were used for matched data, and regular logistic regressions for other data. Comparisons between groups were unweighted.

## Results

### Characteristics of the study sample

Severe mental disorders were less common among participating detainees (46.5%) than non-participating detainees (61.6%), but the difference was not significant after Bonferroni corrections were conducted. Most of the participating detainees and their controls were men (87%) and unemployed (78%). The median age among participating detainees was 31 years (range: 18–69, *IQR*: 25–41.5), 90.5% were suspects of violent crimes and 85% were later sentenced to some sort of incarceration. Further characteristics are given in Table 2.

**Table 2:** Characteristics of participating and non-participating detainees

	Participants (*N* = 200)	Non-participants (*N* = 86)	Statistic	*p*
Median age (Q_3_-Q_1_)	31 (41.5–25)	35.5 (46–27)	U = 1.67	.096
Sex				
Female	26 (13.0%)	8 (9.3%)	*χ* ^2^ = 0.79	.376
Male	174 (87.0%)	78 (90.7%)		
Occupation				
Employed	32 (16.0%)			
Unemployed	156 (78.0%)			
Students	12 (6.0%)			
SMD at unlawful act	92 (46.0%)	53 (61.6%)	χ ^2^ = 5.88	.015
SMD at investigation	93 (46.5%)	53 (61.6%)	χ ^2^ = 5.51	.019
Principal DSM-IV diagnoses				
Mental retardation	12 (6.0%)			
Pervasive developmental disorders	24 (12.0%)			
ADHD	1 (0.5%)			
Substance-related disorders	31 (15.5%)			
Psychotic disorders	57 (28.5%)			
Mood disorders	11 (5.5%)			
Paraphilias	3 (1.5%)			
Adjustment disorder	9 (4.5%)			
Personality disorders	31 (15.5%)			
All others	6 (3.0%)			
None	15 (7.5%)			
Substance-related disorder^a^	74 (37.0%)			
Offence				
Violent crime	181 (90.5%)	76 (88.4%)	χ ^2^ = 0.30	.585
Lethal crime	15 (7.5%)	8 (9.3%)	χ ^2^ = 0.26	.607
Sanction				
Forensic psychiatric care with separate discharge review	73 (36.5%)	48 (55.8%)	FET = 10.03	.051
Forensic psychiatric care without separate discharge review	15 (7.5%)	4 (4.7%)		
Imprisonment	81 (40.5%)	28 (32.6%)		
Probation	27 (13.5%)	6 (7.0%)		
Imprisonment + probation	1 (.5%)	0		
Prosecution dismissed	3 (1.5%)	0		

*Q*_*3*_ *-Q*_*1*_ interquartile range, *U* Mann–Whitney U, *χ* ^*2*^ chi square, *SMD* severe mental disorder, *DSM-IV* Diagnostic and statistical manual of mental disorders, 4^th^ edition, *ADHD* attention deficit hyperactivity disorders, *FET* Fisher’s exact test

^a^Principal or secondary diagnosis

### Missing data

Missing data regarding victimization and health service utilization are reported in Tables 3 and 4. We omitted these data from calculations as they amounted to less than 3%; this allows for complete case analyses [48].

**Table 3:** Violent victimization and health service utilization among detainees and matched controls from the general population

	Detainees *N* = 200 *n* (%) *NA*	Controls *N* = 600 *n* (%) *NA*	Crude risk ratio (95% CI)	Wald	*p*
Violent victimization					
1. Medical attention^a^	33 (16.6) 1	14 (2.4) 12	7.9 [4.0, 15.2]	36.98	<.001
2. Visible injury^b^	63 (31.7) 1	19 (3.2) 12	16.9 [8.6, 32.9]	68.16	<.001
3. No visible injury^c^	47 (23.6) 1	12 (2.0) 12	13.4 [6.8, 26.5]	55.25	<.001
4. Threat	66 (33.3) 2	31 (5.3) 12	8.7 [5.3, 14.4]	70.81	<.001
5. Severe violence (1 + 2)	73 (36.7) 1	32 (5.4) 12	10.1 [6.1, 16.8]	79.31	<.001
6. Violence (1 + 2 + 3)	86 (43.2) 1	41 (7.0) 12	9.3 [5.9, 14.7]	91.54	<.001
7. Any (1 + 2 + 3 + 4)	104 (52.3) 1	65 (11.1) 12	8.2 [5.4, 12.3]	99.51	<.001
Health service utilization					
8. Appointment	95 (47.7) 1	142 (23.7) 1	2.0 [1.4, 2.8]	16.57	<.001
9. Unmet need	84 (42.2) 1	100 (16.7) 2	3.4 [2.4, 4.9]	46.58	<.001

Crude risk ratios are calculated by means of Cox regressions. *NA* missing, *CI* confidence interval

^a^Subjected to violence that required medical attention. ^b^Subjected to violence that caused visible injuries but that did not require medical attention. ^c^Subjected to violence that did not cause visible injuries

**Table 4:** Violent victimization and health service utilization among detainees with severe and non-severe mental disorders

	Severe mental disorder *n* = 92 *n* (%) *NA*	Non-severe mental disorder *n* = 108 *n* (%) *NA*	*OR* (95% CI)	*χ* ^2^	*p*
Violent victimization					
1. Medical attention^a^	10 (11.0) 1	23 (21.3) 0	2.2 [1.0, 4.9]	3.79	.051
2. Visible injury^b^	18 (19.8) 1	45 (41.7) 0	2.9 [1.5, 5.5]	10.93	.001
3. No visible injury^c^	14 (15.4) 1	33 (30.6) 0	2.4 [1.2, 4.9]	6.30	.012
4. Threat	24 (26.7) 2	42 (38.9) 0	1.8 [1.0, 3.2]	3.30	.069
5. Severe violence (1 + 2)	22 (24.2) 1	51 (47.2) 0	2.8 [1.5, 5.2]	11.29	.001
6. Violence (1 + 2 + 3)	29 (31.9) 1	57 (52.8) 0	2.4 [1.3, 4.3]	8.80	.003
7. Any (1 + 2 + 3 + 4)	40 (44.0) 1	64 (59.3) 0	1.9 [1.1, 3.3]	4.64	.031
Health service utilization					
8. Appointment	42 (46.2) 1	53 (49.1) 0	1.1 [0.6–2.0]	0.17	.681
9. Unmet need	35 (38.5) 1	49 (45.4) 0	1.3 [0.8–2.3]	0.97	.326

*NA* missing, *OR* odds ratio, *CI* confidence interval

^a^Subjected to violence that required medical attention

^b^Subjected to violence that caused visible injuries but that did not require medical attention. ^c^Subjected to violence that did not cause visible injuries

### Rates and risk ratios of victimization

Violence or threats during the last twelve months were reported by 52.3% of the detainees and 11.1% of the controls, yielding a risk ratio of 8.2. Detainees had an increased risk of being subjected to any sort of violence or threat, with risk ratios ranging from 7.9 for violence that required medical attention to 16.9 for violence that caused visible injuries (Table 3). Detainees with severe mental disorders were less victimized than detainees with non-severe mental disorders. This applied to any violence or threat (44.0 v. 59.3%), severe violence (24.2 v. 47.2%), violence that did not cause visible injuries (15.4 v. 30.6%), and threats (26.7 v. 38.9%). Odds ratios ranged from 1.8 for threats to 2.9 for violence that caused visible injuries (Table 4).

### Rates and risk ratios of health service utilization

Health service appointments during the past three months were reported by 47.7% of the detainees and 23.7% of the controls, yielding a risk ratio of 2.0. Slightly fewer stated that they had had an unmet need for a health service appointment (42.2 and 16.7%, respectively), but in this case the risk ratio was 3.4 (Table 3). There were no differences between detainees with severe mental disorders and detainees with a non-severe mental disorders.

### Association between violent victimization and health service utilization

Violent victimization was reported by 61.9% of detainees with unmet health care needs and by 45.2% of the remaining detainees, resulting in an odds ratio of 2.0. When violent victimization was restricted to threats, the corresponding proportions were 45.8 and 24.3%, and the odds ratio 2.6. Otherwise, there did not seem to be a significant association between violent victimization and health service utilization among detainees (Table 5).

**Table 5:** Violent victimization among detainees with and without medical appointments and unmet health care needs

	Health service appointment					Unmet health care need				
	Appointment *n* = 95 *n* (%)	No appointment *n* = 104 *n* (%)	*OR* (95% CI)	*χ* ^2^	*p*	Unmet need *n* = 84 *n* = (%)	No unmet need *n* = 115 *n* = (%)	*OR* (95% CI)	*χ* ^2^	*p*
Violent victimization										
1. Medical attention^a^	17 (17.9)	16 (15.4)	1.2 [0.6, 2,5]	0.23	.634	16 (19.0)	17 (14.8)	1.4 [0.6, 2.9]	0.64	.424
2. Visible injury^b^	33 (34.7)	30 (28.8)	1.3 [0.7, 2.3]	0.80	.732	33 (39.3)	30 (26.1)	1.8 [1.0, 3.4]	3.90	.048
3. No visible injury^c^	25 (26.3)	22 (21.2)	1.3 [0.7, 2.6]	0.73	.392	26 (31.0)	21 (18.3)	2.0 [1.0, 3.9]	4.33	.037
4. Threat	38 (40.4)	28 (26.9)	1.8 [1.0, 3.3]	4.05	.044	38 (45.8)	28 (24.3)	2.6 [1.4, 4.8]	9.97	.002
5. Severe violence (1 + 2)	38 (40.0)	35 (33.7)	1.3 [0.7, 2.3]	0.86	.353	37 (44.0)	36 (31.3)	1.7 [1.0, 3.1]	3.40	.065
6. Violence (1 + 2 + 3)	43 (45.3)	43 (41.3)	1.2 [0.7, 2.1]	0.31	.577	40 (47.6)	46 (40.0)	1.4 [0.8, 2.4]	1.15	.284
7. Any (1 + 2 + 3 + 4)	54 (56.8)	50 (48.1)	1.4 [0.8, 2.5]	1.53	.216	52 (61.9)	52 (45.2)	2.0 [1.1, 3.5]	5.42	.020

*OR* odds ratio, *CI* confidence interval

^a^Subjected to violence that required medical attention

^b^Subjected to violence that caused visible injuries but that did not require medical attention

^c^Subjected to violence that did not cause visible injuries

## Discussion

Our study showed that the detainees were significantly more likely to report violent victimization, health service utilization, and unmet health care needs than the controls. Among detainees, those with non-severe mental disorders and unmet health care needs were more likely to report victimization.

### Research implications

To interpret the research implications of this study, its results must be compared with the results of other studies. We restricted our comparisons to studies reporting a one-year prevalence. As we found no relevant research on offenders with mental disorders, we included studies of general psychiatric patients and offenders in general.

Regarding victimization, a Swedish study showed that 20.0% of general psychiatric patients had been subjected to severe violence, 10.2% to violence that required medical attention and 14.5% to violence that caused a visible injury; this study used the same instrument as we did, i.e. SILC [42]. The small number of Swedish studies necessitates international comparisons, although the countries differ in terms of legislation and victimization rates in the general population [49]. Previous research has reported that the one-year prevalence of victimization among general psychiatric patients is 6.4–37.8% in Europe [50–54], 7.1% in Taiwan [55], 17.4% in Ethiopia [56], 17.9–33.8% in Oceania [57, 58], and 7.1–58.6% in the US [59–64]; among offenders it is 18.6% in the Netherlands [65] and 2.8–40% in the US [66, 67]. The studies are not entirely comparable because of different constructs, sites, samples, and methods. However, our work showed a higher prevalence—52.3%—than most other studies. This may indicate that offenders with mental disorders are victimized not only more often than the general population, but also more often than general psychiatric patients and offenders in general.

Most studies on health service utilization among persons with mental disorders focus on consultation owing to mental health problems. Surveys have indicated that the proportion of persons with mental disorders who sought health care services for psychiatric problems during a 12-month period is 25.7% in Europe [68] and 36.0–52.0% in North America [69, 70]. Compared to these numbers, our study showed a proportion in the higher range—47.7%—although the time frame was only three months. However, our study included consultation for both psychiatric and somatic reasons.

Further studies of the group of offenders with mental disorders may add to our understanding. Factors such as diagnoses, concurrent addiction problems, violence risk categories, and type of crimes should be explored concerning their link to victimization and health service utilization. Future research should also incorporate interventions designed to manage victimization and unmet health care needs.

### Clinical and policy implications

As our study indicates that offenders with mental disorders are particularly subjected to violence, society must take measures to prevent their victimization. Violence prevention may be divided into universal, selected, and indicated interventions [11]. That division may be useful also regarding health service utilization.

Universal interventions avert onset of victimization by reducing risk factors from a public health perspective. These interventions are not within the scope of this study as it deals with a certain risk group, but previous research suggests that actions to reduce poverty and addiction [71] and to control the use of firearms [72] may be considered. To facilitate equal health service utilization, the level of prepayment should be high [73].

Selected interventions avert onset of victimization by reducing risk factors in risk groups, in this case offenders with mental disorders. Conceivable actions would be directed psychiatric treatment and addiction treatment. Another action would be founding of staffed accommodation, as there is an association between victimization and homelessness [74]. Health service utilization would be improved by interdisciplinary yet specialized health centers without complicated referral procedures, by specialized outreach teams, and by motivational interventions.

Indicated interventions are actions to reduce harmful consequences of victimization that has already occurred. As our study showed a high rate of victimization among offenders with mental disorders, professionals within social services, health care services, correctional facilities, and the police, should always ask these individuals about victimization and see to it that they obtain requisite health care. Such health care should focus on both somatic and mental aspects of victimization. As this calls for cooperation between different authorities, use of case managers may be considered. Again, health care utilization would be facilitated by interdisciplinary teams and motivational techniques. Hospital-based intervention programs may be another way to reduce violent reinjuries [75].

In our study, there did not seem to be a distinct association between violent victimization and health service utilization. This is another indication of the need of health service improvements for offenders with mental disorders.

### Limitations

There were some drawbacks to this study. As mentioned, matching creates a sample that is not entirely representative of the population. Controls were not asked about forensic psychiatric history and were in consequence not excluded on this basis, but as only a few hundred major forensic psychiatric evaluations are carried out each year this is not likely to have posed a substantial problem. Another limitation is that there appear to be no published studies of the validity of the SILC questionnaire, from which the questions in our study were obtained. The study outcomes relied solely on data obtained from the participants themselves, which posed a risk of recall bias; for example, offenders with severe mental disorders may have had an impaired memory function. Another possible bias may have arisen from the fact that the detainees were interviewed face-to-face whereas the controls were interviewed over the telephone; previous research indicates that telephone respondents may be both more complaisant and suspicious [76]. The time frame regarding unmet health care needs was not uniform for the controls, and the difference between detainees and controls may consequently be larger. Finally, the analysis of association between victimization and health service utilization was limited because it did not include the temporal aspect; there was no data on whether the victimization occurred prior to the health service appointment or afterwards.

## Conclusion

In line with the first three hypotheses, the major findings of this study were that offenders with mental disorders reported more (1) victimization, (2) health service utilization, and (3) unmet health care needs than the general population. For different kinds of violence, the risk ratios ranged from 7.9 to 16.9, confirming that offenders with mental disorders constitute a particularly exposed group. The high risk ratios were present although plausible important confounders were handled by matching, suggesting that the combination of psychiatric disorders and offending history is exceedingly detrimental. These findings tally with the theories of lifestyle, routine activities, and precipitation. Even though the offenders with mental disorders utilized more health services than the controls, their likewise more prevalent experience of unmet health care needs indicates that the offered services are insufficient.

The last three hypotheses were not supported by the results; offenders with severe mental disorders did not report more (4) victimization, (5) health service utilization, and (6) unmet health care needs than offenders with non-severe mental disorders. It may be that non-severe mental disorders, such as substance-related disorders or personality disorders without psychotic symptoms, pose an equal or even larger victimization risk in this population than e.g. psychoses. The present models of health services may also suit offenders with severe mental disorders better, which could explain the study results.

Finally, violent victimization seemed to be associated with unmet health care needs but not actual health service utilization. This indicates that the health services are inadequate for this group.

## Abbreviations

ADHD: Attention deficit hyperactivity disorder
CI: Confidence interval
DSM-IV: Diagnostic and statistical manual of mental disorders, 4^th^ edition
FET: Fisher’s exact test
HCR-20: Historical, Clinical, Risk Management-20
OR: Odds ratio
SILC: Swedish living conditions surveys
SMD: Severe mental disorder

## References

[1] Timmerman IGH, Emmelkamp PMG (2001). The prevalence and comorbidity of axis I and axis II pathology in a group of forensic patients. Int J Offender Ther Comp Criminol.

[2] Belfrage H, Bergman B, Jacks J (1997). Somatic morbidity in patients examined in forensic psychiatry. Int J Law Psychiatry.

[3] Kivimies K, Repo-Tiihonen E, Tiihonen J (2012). The substance use among forensic psychiatric patients. Am J Drug Alcohol Abuse.

[4] Ojansuu I, Putkonen H, Tiihonen J (2015). Mortality among forensic psychiatric patients in Finland. Nord J Psychiatry.

[5] Tabita B, de Santi MG, Kjellin L (2012). Criminal recidivism and mortality among patients discharged from a forensic medium secure hospital. Nord J Psychiatry.

[6] Björk T, Lindqvist P (2005). Mortality among mentally disordered offenders: a community based follow-up study. Crim Behav Ment Health.

[7] Coid J, Kahtan N, Cook A, Gault S, Jarman B (2001). Predicting admission rates to secure forensic psychiatry services. Psychol Med.

[8] Latalova K, Kamaradova D, Prasko J (2014). Violent victimization of adult patients with severe mental illness: a systematic review. Neuropsychiatr Dis Treat.

[9] Elbogen EB, Johnson SC (2009). The intricate link between violence and mental disorder: results from the National Epidemiologic Survey on Alcohol and Related Conditions. Arch Gen Psychiatry.

[10] Degl’ Innocenti A, Hassing LB, Lindqvist AS, Andersson H, Eriksson L, Hanson FH, Möller N, Nilsson T, Hofvander B, Anckarsäter H (2014). First report from the Swedish National Forensic Psychiatric Register (SNFPR). Int J Law Psychiatry.

[11] Krug EG, Dahlberg LL, Mercy JA, Zwi AB, Lozano R, World Health Organization (2002). World report on violence and health.

[12] van Dijk JJM, van Kesteren J, Smit P (2007). Criminal victimisation in international perspective: Key findings from the 2004–2005 ICVS and EU ICS.

[13] Statistics Sweden: Swedish Living Conditions Suveys 2012–2013. In*.*; 2013.

[14] Reiss AJ, Roth JA (1993). Understanding and preventing violence.

[15] Lee BA, Schreck CJ (2005). Danger on the streets. Marginality and victimization among homeless people. Am Behav Sci.

[16] Padgett DK, Struening EL (1992). Victimization and traumatic injuries among the homeless: associations with alcohol, drug, and mental problems. Am J Orthopsychiatry.

[17] Seymour A, Rynearson EK, Seymour A, Murray M, Sigmon J, Hook M, Edmund C, Gaboury M, Coleman G (2002). Substance abuse and victimization. National victim assistance academy textbook.

[18] Pratt TC, Turanovic JJ (2016). Lifestyle and routine activity theories revisited: the importance of “risk” to the study of victimization. Victims Offenders.

[19] Hindelang MJ, Gottfredson MR, Garofalo J (1978). Victims of personal crime: an empirical foundation for a theory of personal victimization.

[20] Cohen LE, Felson M (1979). Social change and crime rate trends: a routine activity approach. Am Sociol Rev.

[21] Wolfgang M. Victim precipitated criminal homicide. J Crim Law Criminol Police Sci. 1957;48(1):1–11.

[22] Cusack KJ, Frueh BC, Brady KT (2004). Trauma history screening in a community mental health center. Psychiatr Serv.

[23] Mueser KT, Goodman LB, Trumbetta SL, Rosenberg SD, Osher F, Vidaver R, Auciello P, Foy DW (1998). Trauma and posttraumatic stress disorder in severe mental illness. J Consult Clin Psychol.

[24] Maniglio R (2009). Severe mental illness and criminal victimization: a systematic review. Acta Psychiatr Scand.

[25] Choe JY, Teplin LA, Abram KM (2008). Perpetration of violence, violent victimization, and severe mental illness: balancing public health concerns. Psychiatr Serv.

[26] Jennings WG, Piquero AR, Reingle JM (2012). On the overlap between victimization and offending: a review of the literature. Aggress Violent Behav.

[27] Silver E, Piquero AR, Jennings WG, Piquero NL, Leiber M (2011). Assessing the violent offending and violent victimization overlap among discharged psychiatric patients. Law Hum Behav.

[28] Desmarais SL, Van Dorn RA, Johnson KL, Grimm KJ, Douglas KS, Swartz MS (2014). Community violence perpetration and victimization among adults with mental illnesses. Am J Public Health.

[29] Sariaslan A, Lichtenstein P, Larsson H, Fazel S (2016). Triggers for violent criminality in patients with psychotic disorders. JAMA Psychiat.

[30] Wood SR (2012). Dual severe mental and substance use disorders as predictors of federal inmate assaults. Prison J.

[31] Blitz CL, Wolff N, Shi J (2008). Physical victimization in prison: the role of mental illness. Int J Law Psychiatry.

[32] Blackwell DL, Lucas JW, Clarke TC (2012). Summary health statistics for U.S. adults: national health interview survey. Vital Health Stat 10.

[33] Devaux M, Lafortune G, Murakami Y, Biondi N, OECD (2014). Unmet health care needs. Health at a Glance: Europe 2014.

[34] Larsen JI, Andersen UA, Becker T, Bickel GG, Bork B, Cordes J, Frasch K, Jacobsen BA, Jensen SO, Kilian R (2013). Cultural diversity in physical diseases among patients with mental illnesses. Aust N Z J Psychiatry.

[35] Reininghaus U, Dutta R, Dazzan P, Doody GA, Fearon P, Lappin J, Heslin M, Onyejiaka A, Donoghue K, Lomas B, et al. Mortality in schizophrenia and other psychoses: a 10-year follow-up of the ӔSOP first-episode cohort. Schizophr Bull. 2014. 10.1093/schbul/sbu138 PMC4393685 25262443

[36] Ringen PA, Engh JA, Birkenaes AB, Dieset I, Andreassen OA (2014). Increased mortality in schizophrenia due to cardiovascular disease - a non-systematic review of epidemiology, possible causes, and interventions. Front Psych.

[37] Fazel S, Baillargeon J (2011). The health of prisoners. Lancet.

[38] Fazel S, Seewald K (2012). Severe mental illness in 33,588 prisoners worldwide: systematic review and meta-regression analysis. Br J Psychiatry.

[39] Fazel S, Danesh J (2002). Serious mental disorder in 23000 prisoners: a systematic review of 62 surveys. Lancet.

[40] Wilper AP, Woolhandler S, Boyd JW, Lasser KE, McCormick D, Bor DH, Himmelstein DU (2009). The health and health care of US prisoners: results of a nationwide survey. Am J Public Health.

[41] Hiday VA, Swartz MS, Swanson JW, Borum R, Wagner HR (2002). Impact of outpatient commitment on victimization of people with severe mental illness. Am J Psychiatry.

[42] Sturup J, Sorman K, Lindqvist P, Kristiansson M (2011). Violent victimization of psychiatric patients: a Swedish case–control study. Soc Psychiatry Psychiatr Epidemiol.

[43] Living Condition Surveys. http://www.scb.se/en/finding-statistics/statistics-by-subject-area/living-conditions/living-conditions/living-conditions-surveys-ulfsilc/. Accessed 7 Mar 2017.

[44] Taylor JM (1986). Choosing the number of controls in a matched case–control study, some sample size, power and efficiency considerations. Stat Med.

[45] Gregg MB (2008). Field epidemiology.

[46] Rose S, Laan MJ (2009). Why match? Investigating matched case–control study designs with causal effect estimation. Int J Biostat.

[47] Statistics Sweden. Definitions of indicators in the ULF/SILC. In*.* Edited by Sweden S; 2013

[48] Harrell FE (2001). Regression modeling strategies : with applications to linear models, logistic regression, and survival analysis.

[49] van Wilsem J (2004). Criminal victimization in cross-national perspective: an analysis of rates of theft, violence and vandalism across 27 countries. Eur J Criminol.

[50] Walsh E, Moran P, Scott C, McKenzie K, Burns T, Creed F, Tyrer P, Murray RM, Fahy T, Group U (2003). Prevalence of violent victimisation in severe mental illness. Br J Psychiatry.

[51] Kamperman AM, Henrichs J, Bogaerts S, Lesaffre EM, Wierdsma AI, Ghauharali RR, Swildens W, Nijssen Y, van der Gaag M, Theunissen JR (2014). Criminal victimisation in people with severe mental illness: a multi-site prevalence and incidence survey in the Netherlands. PLoS One.

[52] Bengtsson-Tops A, Ehliasson K (2012). Victimization in individuals suffering from psychosis: a Swedish cross-sectional study. J Psychiatr Ment Health Nurs.

[53] Fortugno F, Katsakou C, Bremner S, Kiejna A, Kjellin L, Nawka P, Raboch J, Kallert T, Priebe S (2013). Symptoms associated with victimization in patients with schizophrenia and related disorders. PLoS One.

[54] Meijwaard SC, Kikkert M, de Mooij LD, Lommerse NM, Peen J, Schoevers RA, Van R, de Wildt W, Bockting CL, Dekker JJ (2015). Risk of criminal victimisation in outpatients with common mental health disorders. PLoS One.

[55] Hsu CC, Sheu CJ, Liu SI, Sun YW, Wu SI, Lin Y (2009). Crime victimization of persons with severe mental illness in Taiwan. Aust N Z J Psychiatry.

[56] Tsigebrhan R, Shibre T, Medhin G, Fekadu A, Hanlon C (2014). Violence and violent victimization in people with severe mental illness in a rural low-income country setting: a comparative cross-sectional community study. Schizophr Res.

[57] Chapple B, Chant D, Nolan P, Cardy S, Whiteford H, McGrath J (2004). Correlates of victimisation amongst people with psychosis. Soc Psychiatry Psychiatr Epidemiol.

[58] Silver E, Arseneault L, Langley J, Caspi A, Moffitt TE (2005). Mental disorder and violent victimization in a total birth cohort. Am J Public Health.

[59] Pandiani JA, Banks SM, Carroll BB, Schlueter MR (2007). Crime victims and criminal offenders among adults with serious mental illness. Psychiatr Serv.

[60] Brunette MF, Drake RE (1997). Gender differences in patients with schizophrenia and substance abuse. Compr Psychiatry.

[61] Teplin LA, McClelland GM, Abram KM, Weiner DA (2005). Crime victimization in adults with severe mental illness: comparison with the National Crime Victimization Survey. Arch Gen Psychiatry.

[62] Lehman AF, Linn LS (1984). Crimes against discharged mental patients in board-and-care homes. Am J Psychiatry.

[63] Goodman LA, Salyers MP, Mueser KT, Rosenberg SD, Swartz M, Essock SM, Osher FC, Butterfield MI, Swanson J, Committee SHaRSR (2001). Recent victimization in women and men with severe mental illness: prevalence and correlates. J Trauma Stress.

[64] Goodman LA, Thompson KM, Weinfurt K, Corl S, Acker P, Mueser KT, Rosenberg SD (1999). Reliability of reports of violent victimization and posttraumatic stress disorder among men and women with serious mental illness. J Trauma Stress.

[65] Wittebrood K, Nieuwbeerta P (1999). Wages of sin? The link between offending, lifestyle and violent victimization. Eur J Crim Policy Res.

[66] Shaffer JN, Ruback RB. Violent victimization as a risk factor for violent offending among juveniles: Juvenile Justice Bulletin. Rockville: Office of Juvenile Justice and Delinquency Prevention; 2000.

[67] Stephan JJ, Karberg JC, Justice USDo (2003). Census of state and federal correctional facilities, 2000.

[68] Wittchen HU, Jacobi F (2005). Size and burden of mental disorders in Europe--a critical review and appraisal of 27 studies. Eur Neuropsychopharmacol.

[69] Wang PS, Lane M, Olfson M, Pincus HA, Wells KB, Kessler RC (2005). Twelve-month use of mental health services in the United States: results from the National Comorbidity Survey Replication. Arch Gen Psychiatry.

[70] Fleury MJ, Grenier G, Bamvita JM, Perreault M, Caron J (2012). Determinants associated with the utilization of primary and specialized mental health services. Psychiatr Q.

[71] Jewkes R (2002). Intimate partner violence: causes and prevention. Lancet.

[72] Fleegler EW, Lee LK, Monuteaux MC, Hemenway D, Mannix R (2013). Firearm legislation and firearm-related fatalities in the United States. JAMA Intern Med.

[73] Musgrove P, Creese A, Preker A, Baeza C, Anell A, Prentice T (2000). Health systems: Improving performance.

[74] Roy L, Crocker AG, Nicholls TL, Latimer EA, Ayllon AR (2014). Criminal behavior and victimization among homeless individuals with severe mental illness: a systematic review. Psychiatr Serv.

[75] Purtle J, Rich JA, Fein JA, James T, Corbin TJ (2015). Hospital-based violence prevention: progress and opportunities. Ann Intern Med.

[76] Holbrook AL, Green MC, Krosnick JA (2003). Telephone versus face-to-face interviewing of national probability samples with long questionnaires: comparisons of respondent satisficing and social desirability response bias. Public Opin Q.

